# Effect of Intra-Articular Injection of Platelet-Rich Plasma on the Serum Levels of Osteoarthritic Biomarkers in Patients with Unilateral Knee Osteoarthritis

**DOI:** 10.3390/jcm10245801

**Published:** 2021-12-11

**Authors:** Marek Lacko, Denisa Harvanová, Lucia Slovinská, Martin Matuška, Marek Balog, Antónia Lacková, Timea Špaková, Ján Rosocha

**Affiliations:** 1 Department of Orthopedics and Traumatology of Locomotors Apparatus, Faculty of Medicine of P. J. Safarik University, L. Pasteur University Hospital, Trieda SNP 1, 04011 Kosice, Slovakia; martin.matuska@nke.agel.sk (M.M.); marbal82@yahoo.com (M.B.); 2 Associated Tissue Bank, Faculty of Medicine of P. J. Safarik University, L. Pasteur University Hospital, Trieda SNP 1, 04011 Kosice, Slovakia; denisa.harvanova@upjs.sk (D.H.); lucia.slovinska@upjs.sk (L.S.); timea.spakova@upjs.sk (T.Š.); jan.rosocha@upjs.sk (J.R.); 3 Department of Neurology, L. Pasteur University Hospital, Rastislavova 43, 04001 Kosice, Slovakia; antonialackova@gmail.com

**Keywords:** biomarker, knee, osteoarthritis, platelet-rich plasma

## Abstract

Background: The aim of this study is to determine the effect of three doses of intra-articular injection of platelet-rich plasma (PRP) into the osteoarthritic (OA) knee joint on the functional status and on the changes in the levels of specific OA biomarkers in blood serum. Methods: Forty patients with unilateral primary knee osteoarthritis were enrolled in this single center, prospective clinical trial. For each patient, three intra-articular PRP injections were administered one week apart. Clinical and laboratory assessment was performed before the first PRP injection (baseline), and 3 months after the third PRP application (3-month follow up). Pain in the affected knee joint was assessed with the Visual Analog Scale for Pain (VAS). Change in clinical status was evaluated with the Western Ontario and McMaster Universities Arthritis Index Questionnaire (WOMAC). Concentrations of 19 biomarkers (EGF, Eotaxin, FGF-2, GRO, IL-10, IL-1RA, IL-8, IP-10, MCP-1, PDGF-AB/BB, RANTES, MMP-3, MMP-13, Collagen type 2, BMP-2, TIMP-1, TIMP-2, TGF beta 1, and COMP) in the serum of studied patients were quantified. Results: At 3-month follow up, there was a significant decrease in the VAS score and significant improvement in the WOMAC score. There was a significant decrease in the levels of Eotaxin, MCP-1, MMP-1, IL-10, EGF, PDGF-AB/BB, TGF- β1 compared to baseline levels. A significant increase in markers BMP-2, COMP, Collagen type 2 and GRO was found at the same time point. There was no significant change in the concentrations of other biomarkers (FGF-2, IL-1RA, IL-8, IL-10, MMP-3, RANTES, TIMP-1, TIMP-3). Conclusions: We found an increase in specific pro-anabolic and anti-inflammatory biomarkers with a concomitant decrease in pro-inflammatory biomarkers at 3 months after three intra-articular applications of PRP. Significant improvement in VAS and WOMAC scores was observed. Treatment with PRP may be an effective therapeutic option with anti-inflammatory and regenerative potential in patients with primary knee OA.

## 1. Introduction

Osteoarthritis (OA) is the most common degenerative joint disease with a pronounced social impact. OA represents a heterogeneous group of diseases that develop in distinct, overlapping patterns of joint symptoms associated with loss of articular cartilage, osteophyte formation, subchondral sclerosis, and synovial inflammation [1]. The pathophysiology of OA is based on the interaction between cartilage, bone, and synovial tissue, resulting in a vicious cycle of inflammation and cartilage breakdown. The balance between catabolic and anabolic factors in osteoarthritic cartilage is disturbed, with a shift toward catabolic events [2,3].

Treatment of degenerative cartilage disease is difficult because the ability of hyaline cartilage to regenerate is limited due to the lack of blood vessels and nerves. Various medical treatments are widely available, but none are able to stop the progression of the disease or restore the degenerated cartilage. Biological therapies such as platelet-rich plasma have shown promise for treating many diseases, including OA.

Platelet-rich plasma (PRP) is an autologous product of highly concentrated platelets dispersed in a small volume of plasma. The therapeutic potential of PRP is based on the rich supply of anabolic growth factors and anti-inflammatory cytokines in platelets which induce cell proliferation, migration, differentiation, angiogenesis, and extracellular matrix synthesis [4]. PRP may alter the milieu of the osteoarthritic joint, by adding a number of anti-inflammatory and pro-anabolic cytokines that promote regeneration. Recent meta-analyzes and systemic reviews have shown that treatment with PRP can improve clinical outcomes in patients with knee OA, but there is insufficient objective evidence for the anti-inflammatory and regenerative efficacy of PRP in osteoarthritic joints [5,6,7].

### Aim of the Study

The aim of this study is to determine the effect of three doses of intra-articular injection of PRP into the osteoarthritic knee joint on the changes in the levels of specific OA biomarkers in human serum. We hypothesized that PRP has an anti-inflammatory and pro-anabolic effect on cartilage degeneration. The aim was extended to investigate the clinical effect of PRP on the functional status of the osteoarthritic knee.

## 2. Materials and Methods

The study was designed as a single center, prospective clinical trial. Forty patients with unilateral primary knee osteoarthritis diagnosed according to the American College of Rheumatology classification criteria for knee OA [8] were enrolled between June 2020 and August 2021.

All patients had a history of chronic (lasting at least 6 months) moderate to severe pain (4 to 10 points on the visual analog pain scale) in the affected knee.

Exclusion criteria were: secondary knee OA, symptomatic OA of another joint, age over 65 years, body mass index over 35 kg/m^2^, intra-articular injection of steroids or hyaluronic acid in the last 6 months before the PRP injection, infectious diseases, systemic diseases (severe cardiovascular disease, diabetes mellitus, rheumatoid arthritis, haematologic disorders, and malignancy) or patients under immunosuppression, anticoagulant or antiaggregant therapy. Non-steroidal anti-inflammatory drugs should be avoided 2 weeks before the first PRP injection and at least 3 months after the third PRP application.

### 2.1. Study Approval

The study protocol was approved by the local ethics committee (registration number: 2020/EK/02010). Written informed consent was obtained from each patient prior to participation in this study. The study was conducted in accordance with the principles of the Declaration of Helsinki.

### 2.2. Outcome Measures

Clinical assessment was performed before the first PRP injection, and 3 months after the third PRP application. All patients were assessed for pain, stiffness, and functional status using the Western Ontario and McMaster Universities Arthritis Index Questionnaire [9]. Change in pain in the affected knee joint was assessed using the Visual Analog Scale for Pain (VAS; 0 = no pain, 10 = most severe pain imaginable; [10]).

### 2.3. PRP Preparation and Administration

The PRP preparation protocol was based on the previous study evaluating the efficacy and safety of PRP treatment in patients with osteoarthritis [11]. The blood sample (27 ml of venous blood) was drawn into three 10-ml vacutainer tubes (S-Monovette, Sarstedt) containing either 1 mL 0.106 M sodium citrate. The blood sample was then centrifuged for 15 min at 3200 rpm at 20 °C (Labofuge 400R, Heraeus, Hanau, Germany). The buffy coat layer together with the plasma layer was collected and centrifuged for a further 10 min at 1500 rpm to separate the leukocytes. The plasma layer was collected, and the third centrifugation step at 3200 rpm for 10 min was performed to obtain a two-part plasma: the upper part consisted of platelet-poor plasma and the lower part consisted of PRP. The platelet-poor plasma was discarded first to avoid mixing with the PRP. The tubes were shaken for 30 s to suspend the platelets. The buffy coat layer consisting of blood platelets was then carefully aspirated into a syringe in a volume of 3 mL of plasma and used for the intra-articular injection within 30 min. All open procedures were performed in a high-efficiency, particle-filtered, laminar flow cabinet class II.

Platelet and leucocyte levels in the PRP product were not analyzed prior to injection, as these values had already been examined in the previous study [11]. The PRP products were not activated prior to injection, as platelets are known to be activated when they come into contact with collagen tissue.

For each patient, three PRP injections were administered one week apart. The PRP products were injected intra-articularly through a superolateral port. The use of non-steroidal anti-inflammatory drugs was not allowed during the study period. Patients were only allowed to use paracetamol and cold packs as analgesics.

### 2.4. Blood Sampling

Approximately 5 ml of peripheral venous blood was collected from each patient by sterile venipuncture before the first injection of PRP (baseline) and 3 months after the third injection of PRP (3-month follow up). The blood was allowed to clot naturally for 30 min and then centrifuged at 4000 rpm for 10 minutes at 20 °C. The serum was separated and aliquots were stored at −80 °C before following analysis.

### 2.5. Multiplex Assay for Cytokine/Chemokine and Matrix Metalloproteinase Quantification

Concentrations of 13 biomarkers (EGF, eotaxin, FGF-2, GRO, IL-10, IL-1RA, IL-8, IP-10, MCP-1, PDGF-AB/BB, RANTES, MMP-3, MMP-13) were quantified in duplicate for each sample/patient using MILLIPLEX^®^ Assays (Merck KGaA, Darmstadt, Germany) according to the manufacturer’s protocol and MAGPIX Luminex platform. xPONENT software version 4.2 for MAGPIX (Luminex Corporation, Austin, TX, USA) and Bio-Plex Manager 6.1 (Bio-Rad Laboratories, Hercules, CA, USA) were used for data analysis. Once standard curves were generated, the concentrations for each sample were interpolated using a 5-parameter curve fitting equation and expressed in pg/mL.

### 2.6. Enzyme-Linked Immunosorbent Assay (ELISA) Measurement of Human Collagen Type 2, BMP2, TIMP1, TIMP2, TGF Beta 1 and COMP

The concentrations of the target proteins in the serum of studied patients were determined using the Collagen type 2 kits (MyBioSource, San Diego, CA, USA), BMP-2, TIMP-1, TIMP-2, TGF beta 1, and COMP ELISA kits (Abcam, Cambridge, UK) according to the manufacturer’s protocols and recommendations. These kits used the double antibody sandwich ELISA technique. The optical density value of the target biomarker in the sample was determined at 450 nm using a microplate reader (TriStar LB941, Berthold Technologies, Bad Wildbad, Germany). Then the standard curve was constructed using a 5-parameter logistic curve fitting method and the protein concentrations of the tested samples were calculated according to the manufacturer’s instructions.

### 2.7. Statistical Analysis

The descriptive statistics (mean, standard deviation) for the continuous study variables were calculated. The change in WOMAC and VAS scores and biomarker serum concentrations were evaluated using an independent Student t-test. If the normality test failed, the Mann-Whitney test was used. Statistical significance was defined with a significance level of *p* < 0.05. SigmaPlot version 12.5 (Systat Software, Inc., San Jose, CA, USA) was used for statistical analyzes.

## 3. Results

Four patients were excluded from the study: the contralateral knee became symptomatic in two patients and two patients had an acute respiratory infection. Thirty-six patients completed the final 3-month follow-up. The demographics of the patients enrolled in the study are shown in Table 1.

### 3.1. Clinical Outcomes

At the 3-month follow-up, there was a statistically significant decrease in the VAS score (*p* < 0.001). There was also a significant improvement in the WOMAC total score (*p* < 0.001) and in the pain, stiffness and function subscores 3 months after the third PRP injection. The changes in VAS and WOMAC scores are shown in Table 2.

Five patients experienced a transient increase in knee pain and swelling that resolved within 2 days. No further complications were observed.

### 3.2. Laboratory Outcomes

**Figure 1:**
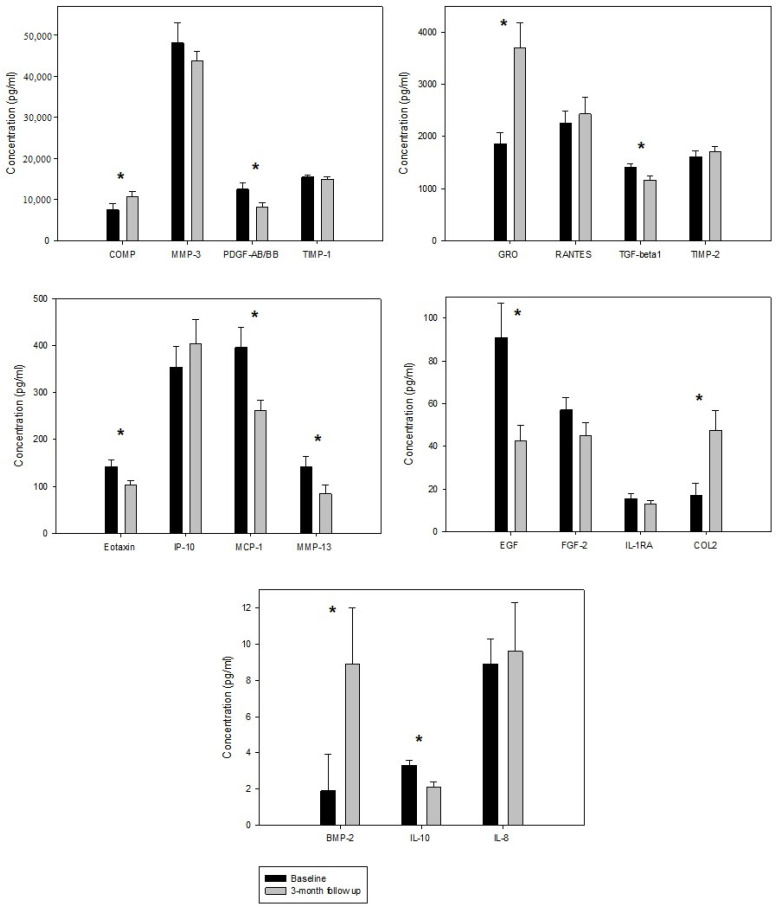
Mean concentration of biomarkers before and 3 months after the treatment with PRP. Error bars indicate standard deviation; differences were analyzed using Student’s *t*-test; * *p* < 0.05.

After follow-up period of 3 months, there was a significant decrease in the levels of pro-inflammatory markers Eotaxin (*p* = 0.039), MCP-1 (*p* = 0.007), MMP-13 (*p* = 0.005), anti-inflammatory biomarker IL-10 (*p* = 0.002) and growth factors EGF (*p* = 0.030), PDGF-AB/BB (*p* = 0.027), TGF- β1 (*p* = 0.026) compared to baseline levels. In addition, there was a statistically significant increase in markers of the cartilage formation (pro-anabolic markers) BMP-2 (*p* = 0.011), COMP (*p* = 0.046), Collagen type 2 (*p* = 0.022) and the anti-inflammatory biomarker GRO (*p* = 0.002) at the same time point. There was no significant change in the blood concentrations of other biomarkers FGF-2 (*p* = 0.119), IL-1RA (*p* = 0.485), IL-8 (*p* = 0.537), IP-10 (*p* = 0.632), MMP-3 (*p* = 0.630), RANTES (*p* = 0.995), TIMP-1 (*p* = 0.240), TIMP-3 (*p* = 0.485). The biomarker levels of the studied patients before and after PRP treatment are shown in Table 3 and Figure 1.

## 4. Discussion

PRP is used in clinical practice for the treatment of degenerative joint lesions in osteoarthritis, tendinitis, and other musculoskeletal conditions, although we have limited information on its mechanism of action. There is no consensus on the efficacy of PRP treatment. Several meta-analyzes and systematic reviews have shown that after the treatment with PRP improvement of clinical outcomes in patients with knee OA can be expected, but different PRP regimens are used for treatment [5,6,7]. We used 3 injections based on the results of the previous study [11] and found that the use of PRP significantly reduced VAS and improved WOMAC scores at 3-month follow-up, confirming the efficacy of PRP in the treatment of OA.

Inflammation of the synovial membrane, which occurs in both early and late stages of OA, is accompanied by changes in the adjacent cartilage [12]. The breakdown of cartilage continues the inflammatory process and leads to further degradation of the cartilage. A higher level of inflammation is associated with more intense pain. The therapeutic goal in OA should be to break this vicious cycle, inhibit the inflammatory mediators, reduce the mediators of cartilage degradation and stimulate the formation of new cartilage. In addition to treatment with PRP, there are competing anti-inflammatory interventions, such as nonsteroidal anti-inflammatory drugs, steroid injections, and low-level laser therapy. They improve function and pain in the short term, but have no regenerative potential in OA joints. It is possible to use biomarkers in serum to monitor response to knee OA therapy. In this study, we evaluated serum biomarkers identified by OARSI (Osteoarthritis Research Society International) using the BIPED (Burden of Disease, Investigative, Prognostic, Efficacy of the intervention, and Diagnostic) classification [13,14]. Three months after the treatment with three PRP injections, we found significantly reduced levels of the pro-inflammatory biomarkers Eotaxin, MCP-1, and MMP-13. This finding may indicate that the injection of PRP has an anti-inflammatory effect. At the same time, we found a statistically significant decrease in the serum concentration of the growth factors EGF, PDGF-AB/BB, TGF-β1, and the anti-inflammatory biomarker IL-10. This could be the result of an overall suppression of the inflammatory process by PRP. In addition, PRP caused a significant increase in serum levels of pro-anabolic biomarkers such as COMP, BMP-2, and Collagen type 2. These results may suggest that the use of PRP is likely effective in cartilage formation. We did not detect any significant changes in serum levels of pro-inflammatory biomarkers MMP-3, RANTES and anti-inflammatory markers TIMP-1 and TIMP-2, which could be due to a long time interval, and these biomarkers showed no activity after 3 months. Because these biomarkers act directly in osteoarthritic joints, assessment of their concentration in synovial fluid is probably more appropriate.

There is a lack of literature reporting the effect of PRP injection on OA blood serum biomarkers. We found only four in vivo human studies on this topic [15,16,17,18].

Mariani et al. [15] performed an analysis of the local and systemic effects induced by three injections of leukocyte-rich PRP. Plasma and synovial fluid were analyzed for the presence of pro- and anti-inflammatory cytokines (IL-1, IL-4, IL-6, IL-8, IL-10, IL-13, IL-17) and growth factors (b-FGF, HGF, PDGF-AB/BB) in 36 patients 2, 6, and 12 months after the treatment with PRP. They found that administration of PRP did not result in significant changes in cytokine concentrations in synovial fluid or plasma, regardless of the time points studied.

Fawzy et al. [16] reported a significant decrease in a serum biomarker of cartilage degradation, Collagen 2-1, over a 3-month period in 60 patients after an injection of PRP. The peptide Collagen 2-1 and its nitrated form, are located in the triple helix of the type 2 collagen molecule and are used as biomarkers of collagen degradation [19]. In our study, we determined the change in the concentration of Collagen type 2 (COL2), which is the major protein component and specific to hyaline cartilage. Its degradation is a main feature of osteoarthritis. We found a significant increase in the average concentration of Collagen type 2, suggesting that PRP plays a role in cartilage formation.

Cartilage oligomeric matrix protein (COMP) is another biomarker studied that is thought to reflect cartilage degradation. The results of some relevant studies show a positive correlation between COMP and the stage of OA [20]. Other studies even found no correlation at all between COMP and the presence of OA [21]. However, no standardized values for the index have been established yet. Kuculumet et al. [17] demonstrated that injections of PRP had no effect on the concentration values of COMP. On the other hand, our results were in agreement with the study of Lychagin et al. [18] who observed a significant increase in COMP concentration associated with significant clinical improvement in patients with chondral lesions treated with PRP injections. They hypothesized that an increase in serum COMP levels reflects not only the progression of joint destruction, but also the occurrence of positive cartilage turnover.

Bone morphogenetic protein 2 (BMP-2) has been suggested as a tool for cartilage repair and a stimulant of chondrogenesis [22]. BMP-2 is rarely present in healthy cartilage, whereas it is highly expressed in osteoarthritis. BMP-2 can induce chondrogenesis in human mesenchymal stem cells in vitro. Ianey Davidson et al. [23] confirmed that BMP activity appears to be involved in cartilage repair and replacement of damaged matrix molecules. We found an increase in BMP-2 levels after the treatment with PRP, suggesting the pro-anabolic effects of PRP. To our knowledge, no comparable results have been published yet.

The conflicting evidence on the biological effects of PRP in clinical trials is likely the result of an unclear definition and lack of standardization of PRP preparation and treatment. Up to date, there is no consensus on the optimal preparation, composition, amount, and activation of PRP, which leads to different contents and properties of PRP products. The cause of contradicting results in the literature could also be the heterogeneous and multifactorial nature of OA itself, as well as the different inflammatory profiles in subtypes of the OA that affect the entire joint structures (cartilage, subchondral bone, ligaments, synovial membrane, and joint capsule). Another reason may be age and sex-related changes in the immune system associated with differential production of cytokines [24]. Although numerous clinical studies suggest that PRP may be a promising treatment for OA, the mechanism of action to improve cartilage repair is not yet clear.

### Limitation

This study has several limitations. The first is the lack of quantitative analysis of the PRP product, although blood cells content was demonstrated in our previous study. There are several studies on OA biomarkers, but there is still no consensus on which biomarker can be used for diagnosis, prognosis, prediction, or response to the treatment. For this reason, the present study examined a relatively wide range of serum markers included in the OARS BIPED classification. Physical activity and circadian rhythm may also influence biomarker levels. To avoid this limitation, blood samples were collected before 10 am, and before breakfast. Another limitation is the lack of a control group of healthy, age-, and sex-matched subjects.

## 5. Conclusions

Current treatments for OA are palliative and focus on symptom relief, but are unable to halt disease progression or cure it. An increase in specific pro-anabolic and anti-inflammatory biomarkers with a concomitant decrease in pro-inflammatory biomarkers and a significant improvement in VAS and WOMAC scores at 3 months after three intra-articular applications of PRP suggest that treatment with PRP can be used as an effective therapeutic option with anti-inflammatory and pro-regenerative potential in patients with primary knee OA. Further research is needed to identify the most appropriate biomarkers for assessing the diagnosis of osteoarthritis, response to treatment, to understand the mechanism of action of PRP in osteoarthritic joints, and to optimize and standardize the formulations of PRP.

## Figures and Tables

**Table 1:** Characteristics of the studied patients.

Variable	OA Group (*n* = 36)
Age (years)	53.4 (±7.7)
Female/Male	22/14
BMI (kg/m^2^)	29.1 (±3.4)
KL grade of OA (I/II/III/IV)	5/19/12/0

*n*: Number; BMI: Body Mass Index; KL: Kellgren-Lawrence grading scale; OA: osteoarthritis; ±: standard deviation.

**Table 2:** VAS and WOMAC index variables at baseline and follow up in patients with unilateral primary knee osteoarthritis.

Variables	Baseline	3-Month Follow Up	*p* Value
VAS	6.5 (±1.4)	3.3 (±1.8)	*p* < 0.001
WOMAC			
Pain	8.8 (±3.1)	3.6 (±2.4)	*p* < 0.001
Stiffness	2.9 (±2.1)	1.2 (±1.5)	*p* < 0.001
Function	23.9 (±9.6)	13.2 (±8.4)	*p* < 0.001
Total	32.8 (±11.9)	18.1 (±11.6)	*p* < 0.001

Values are expressed as mean, with a standard deviation in parentheses; *p* determined with Student’s *t*-test; VAS: Visual analog scale of pain; WOMAC: Western Ontario and McMaster Universities Osteoarthritis Index.

**Table 3:** Summary of biomarker levels in the serum.

Biomarker	Baseline	3-Month Follow Up	*p* Value
BMP-2	1.9 (±2.0)	8.9 (±3.1)	*p* = 0.011
COMP	7545.1 (±1428.4)	10,716.3 (±1174.4)	*p* = 0.046
EGF	90.9 (±16.2)	42.6 (±7.4)	*p* = 0.030
Eotaxin	141.6 (±14.1)	103.1 (±9.9)	*p* = 0.039
FGF-2	57.1 (±5.8)	45.2 (±5.9)	*p* = 0.119
GRO	1860.4 (±213.6)	3708 (±477.7)	*p* = 0.002
IL-10	3.3 (±0.3)	2.1 (±0.3)	*p* = 0.002
IL-1RA	15.5 (±2.2)	13.1 (±1.7)	*p* = 0.485
IL-8	8.9 (±1.4)	9.6 (±2.7)	*p* = 0.537
IP-10	354.9 (±42.8)	404 (±50.5)	*p* = 0.632
Collagen 2	17.1 (±5.6)	47.4 (±9.5)	*p* = 0.022
MCP-1	396.1 (±43.4)	262.7 (±21.6)	*p* = 0.007
MMP-13	141.7 (±22.1)	84.2 (±19.6)	*p* = 0.005
MMP-3	48,084.3 (±4858.3)	43,737.8 (±2298.4)	*p* = 0.630
PDGF-AB/BB	12,513.9 (±1501.2)	8287.7 (±923.8)	*p* = 0.027
RANTES	2250.3 (±240.4)	2434.3 (±313.2)	*p* = 0.995
TGF-β1	1402.4 (±74.1)	1165.8 (±73.4)	*p* = 0.026
TIMP1	15,497.7 (±542.8)	14,958.9 (±568.2)	*p* = 0.240
TIMP2	1612.7 (±107.0)	1710.2 (±97.2)	*p* = 0.252

Values are expressed as mean, with a standard deviation in parentheses; *p* determined with Student’s *t*-test.

## Data Availability

The data presented in this study are available on request from the corresponding author. The data are not publicly available due to personal data protection.
